# CD47-ligation induced cell death in T-acute lymphoblastic leukemia

**DOI:** 10.1038/s41419-018-0601-2

**Published:** 2018-05-10

**Authors:** Pascal Leclair, Chi-Chao Liu, Mahdis Monajemi, Gregor S. Reid, Laura M. Sly, Chinten James Lim

**Affiliations:** 1 Department of Pediatrics, University of British Columbia, Vancouver, BC Canada V5Z 4H4; 2 Department of Medicine, University of British Columbia, Vancouver, BC Canada V5Z 4H4; 3 Michael Cuccione Childhood Cancer Research Program, B.C. Children’s Hospital Research Institute, Vancouver, BC Canada V5Z 4H4

## Abstract

CD47 is a cell-surface marker well recognized for its anti-phagocytic functions. As such, an emerging avenue for targeted cancer therapies involves neutralizing the anti-phagocytic function using monoclonal antibodies (mAbs) to enhance tumour cell immunogenicity. A lesser known consequence of CD47 receptor ligation is the direct induction of tumour cell death. While several mAbs and their derivatives with this property have been studied, the best characterized is the commercially available mAb B6H12, which requires immobilization for induction of cell death. Here, we describe a commercially available mAb, CC2C6, which induces T-cell acute lymphoblastic leukemia (ALL) cell death in soluble form. Soluble CC2C6 induces CD47-dependent cell death in a manner consistent with immobilized B6H12, which is characterized by mitochondrial deficiencies but is independent of caspase activation. Titration studies indicated that CC2C6 shares a common CD47-epitope with B6H12. Importantly, CC2C6 retains the anti-phagocytic neutralizing function, thus possessing dual anti-tumour properties. Although CD47-ligation induced cell death occurs in a caspase-independent manner, CC2C6 was found to stimulate increases in Mcl-1 and NOXA levels, two Bcl-2 family proteins that govern the intrinsic apoptosis pathway. Further analysis revealed that the ratio of Mcl-1:NOXA were minimally altered for cells treated with CC2C6, in comparison to cells treated with agents that induced caspase-dependent apoptosis which alter this ratio in favour of NOXA. Finally, we found that CC2C6 can synergize with low dose chemotherapeutic agents that induce classical apoptosis, giving rise to the possibility of an effective combination treatment with reduced long-term sequelae associated with high-dose chemotherapies in childhood ALL.

## Introduction

Acute lymphoblastic leukemia (ALL) is the most commonly occurring childhood cancer, accounting for 25% of patients under 15 years old. ALL is highly treatable, achieving a 5-year disease-free rate approaching 90%^1^. Disease treatment is stratified into low and high-risk therapies, with vincristine, corticosteroids, and asparaginase included in both, while anthracyclines are reserved for high-risk patients given their elevated toxicity^1^. The long-term effects of high-dose chemotherapeutics carry an extensive burden of morbidity that may be fatal^2^. For example, doxorubicin is associated with increased risk of cardiomyopathy and secondary neoplasms, while neurotoxicity is associated with vincristine^2,3^. Due to the high treatment success rates for childhood ALL, the agents used in conventional chemotherapy have remained largely unchanged for three decades^4^. However, there remains a need for novel therapeutics, especially ones with reduced systemic toxicities, for improved long-term treatment outcomes and for those experiencing relapse with acquired chemoresistance.

CD47 is a 5-time spanning membrane receptor involved in various functions, including cell adhesion, T-cell activation, inhibition of NO-signaling, and, via its counter-receptor, SIRPα, inhibition of phagocytosis^5–7^. Increased CD47-expression has been observed in a variety of tumour cells and considered an adverse prognostic factor^8^. It is also the target of therapeutic intervention, achieved by antigen receptor neutralization using antibodies^9–13^. One aspect of CD47-mediated signaling that remains poorly exploited is its role in promoting cell death. Cell death can be triggered by CD47-ligation with certain antibodies, however, the most studied monoclonal antibody used to date, mAb B6H12, achieves this activity in immobilized form, a fact limiting its usefulness^14,15^. Several CD47-antibodies that induce cell death in the soluble state have been described (clones 1F7, Ad22 and MABL)^14,16,17^, however these commercially unavailable reagents have limited their exploration in understanding CD47-induced cell death pathways.

Apoptosis is a cell physiological program that enables the controlled removal of cells without triggering an immune response. Specifically, the intrinsic apoptosis pathway is enabled by mitochondria disruption resulting from dysregulation of the delicate balance of Bcl-2 family proteins, namely, the pro-survival Mcl-1, and its regulator, the pro-apoptotic NOXA^18^. Although caspase activation is a hallmark of apoptosis, evidence for caspase-independent cell death exists, including heat shock-induced cell death^19^, activities mediated by granzymes^20^, and CD47-receptor ligation^21,22^. Interestingly, despite being caspase-independent, many of these pathways still have phenotypes associated with classical apoptosis. For example, CD47-mediated cell death is associated with increased reactive oxygen species (ROS), disruption of mitochondrial membrane potential, and decreased ATP.

Given that most chemotherapeutic agents induce apoptosis via the intrinsic, caspase-dependent pathway^23^, we hypothesized that CD47-mediated cell death could complement the effects of chemotherapy since it employs a caspase-independent pathway. Here, we describe the cell death-inducing properties of CC2C6, a commercially available CD47-mAb, in T-lymphoblasts, and characterized its activity with regards to regulation of Mcl-1 and NOXA protein levels. In addition, CC2C6 potentiated the effects of commonly used chemotherapeutics at sub-optimal concentrations, including a synergistic effect when used in combination with the novel therapeutic, honokiol. Our results lend support for continued evaluation of the potentially multi-therapeutic benefits of targeting CD47 as a form of tumour immuno-therapy.

## Methods and materials

### Cells and reagents

Jurkat T-lymphoblasts (clone E6-1) were purchased form the American Type Culture Collection. Dr. Weng provided the T-ALL cell lines THP-6, SUP-T1, DND-41, Peer, BE-13 and Karpas. Dr. Roberts provided the CD47-decifient Jurkat cell line, JinB8^24^. All cells were maintained at 37 °C, 5% CO_2_, in cRPMI (cRPMI is RPMI 1640 supplemented with 10% fetal bovine serum (Invitrogen), non-essential amino acids (Invitrogen), and penicillin-streptomycin (Gibco)). Where indicated, serum-free RPMI contains 1% bovine serum albumin (BSA). The primary T-ALL, BD-67, was as described previously^25^. Cells harvested from spleens of xenografted mice were maintained in vitro up to 7 days in StemSpan SFEMII (StemCell Technologies). Chemotherapeutics used were doxorubicin and vincristine (Sigma-Aldrich), bortezomib (cell signaling) and honokiol (Enzo). All other reagents were from Sigma unless otherwise stated.

### Plasmids, transfections and CRISPR gene modifications

Guide sequence 5′CTGGTAGCGGCGCTGTTGCT3′ targeting exon 1 of CD47 was inserted into the pX330 CRISPR-Cas9 plasmid^26^. Jurkat cells were nucleofected (Amaxa Nucleofector II, Lonza) with this plasmid, cultured for 1 week, and clonally sorted for CD47^−/−^ cells (named JC47 for Jurkat CRISPR CD47) by flow cytometry. Loci targeting in candidate clonal populations was confirmed by sequencing the genomic amplicon generated by polymerase chain reaction (PCR) using the primers: 5′GACAGGAACGGGTGCAATGA3’ and 5′TAATTTTTGCGCGAGGTGCG3′. Analyses were performed using CLC Main Workbench (CLC Bio) by alignment of CRISPR mutants to the parental amplicon sequence.

The expression construct for CD47 isoform 4 (pKS336-hCD47iso4) was provided by Dr. Ohdan (University of Hiroshima)^27^. CD47iso1 and iso2, with shorter cytoplasmic domains, were generated from CD47iso4 using PCR, and re-inserted into the pKS336 backbone. CD47^−/−^ cells were nucleofected and serially sorted by flow cytometry to obtain clonal populations of CD47-expressing cells.

### Antibodies

The following primary antibodies were used in this study: CC2C6 (Biolegend), B6H12 (BD Pharmingen) and BRIC126 (Santa Cruz Biotechnology) are α-CD47 monoclonals; Mcl-1, PARP-1 (Santa Cruz Biotechnology); NOXA (Cell Signaling); 9F10 (ITGA4), P84 (SIRPα), GAPDH, and Alexa Fluor 647-labelled F4/80 (Biolegend). Secondary antibodies used were Dylight488, Dylight633, Dylight680 and DyLight800 conjugates of goat-anti-mouse, and Dylight800 conjugated goat-anti-rabbit (all from Thermo Fisher).

### Flow cytometry

Analytical flow cytometry was conducted on the Accuri, FacsCanto, LSRFortessa X-20, or the LSRII instruments (BD Biosciences). Cell sorting was conducted on the FacsAria (BD Biosciences) at the BCCHRI Flow Core. Post-acquisition analysis was conducted using FlowJo (Tree Star).

### Apoptosis assays

Cells were washed in phosphate-buffered saline (PBS) and re-suspended in cRPMI. Cells were transferred as 1.0 mL aliquots to 24-well dishes for replicate treatments, and incubated at 37 °C, 5% CO_2_ for 2 h (unless otherwise indicated) with and without antibodies. In some experiments, antibodies, chemotherapeutics, or other pharmaceuticals were added at the indicated concentrations. For intracellular calcium experiments, cells were pre-labelled with Fluo4-AM (Life Technologies) according to manufacturer’s protocol. Cells were pre-treated with BAPTA-AM, while EGTA is added to media, to chelate intracellular and extracellular calcium, respectively. Following incubation, cells were harvested and washed in PBS, re-suspended in 1 × binding buffer containing Annexin V (Cy5-labelled from BD Pharmingen or made in-house labelled with FITC) with or without propidium iodide (PI, 2 μg/mL final) and analyzed by flow cytometry. ROS was analyzed using MitoSox Red reagent (Molecular Probes, Invitrogen) as suggested by the manufacturer, and mitochondrial membrane potential was assessed using 40 nM 3,3′-dihexyloxacarbocyanine iodide (DiOC_6_). Caspase activation was assessed using Caspase Glo3/7 (Promega) as suggested by the manufacturer.

### Blocking assay with CC2C6

PBS-washed cells were incubated with varying B6H12 concentrations in 1% BSA/PBS for 20 min at 4 °C. After washing, cells were labelled with CC2C6-FITC in 1% BSA/PBS for 20 min at 4 °C. CC2C6-FITC binding was assessed by flow cytometry, and B6H12 blockade of CC2C6-FITC binding was calculated as follows: 100*[(GMFI of CC2C6-FITC with B6H12)/(GMFI of CC2C6-FITC without B6H12)]; where GMFI is the geometric mean fluorescence intensity.

### Protein immunoblotting assays

Cell lysates were prepared in RIPA lysis buffer (50 mM Tris, pH8, 150 mM NaCl, 1% Triton-X, 0.5% sodium deoxycholate, 0.1% SDS, 1 mM EDTA, and protease inhibitor cocktail (Roche)), separated by SDS-PAGE and transferred to nitrocellulose membranes using the Transblot Turbo Transfer System (Bio-Rad). Membranes were labelled with the indicated primary and secondary antibodies and, imaged and quantified on the LI-COR Odyssey. Where applicable, quantification of protein bands of interest were normalized relative to GAPDH levels.

### Phagocytosis experiments

Bone marrow derived macrophages were prepared from 8-week-old C57BL/6 mice, as described previously^25^. For phagocytosis assays, cultured macrophages were lifted using Cell Dissociation Buffer as per manufacturer’s instructions (Gibco, Thermo-Scientific) and re-suspended in complete IMDM at 5 × 10^5^ cells/mL. Macrophages were spun down and re-suspended in blank IMDM for 1 h prior to co-incubating with target cells. Jurkat target cells were labeled with CellTracker*™* Green (Invitrogen) according to manufacturer’s instructions and then incubated with or without 7 μg/mL B6H12 or CC2C6 for 2 h at 37 °C. Cells were washed twice with PBS and re-suspended in serum-free IMDM. Phagocytosis assays were initiated by mixing 2.5 × 10^5^ macrophages with 7 ×  10^5^ Jurkat cells in 24-well plates for 2 h in serum-free IMDM at 37 °C. Murine macrophages were subsequently stained with F4/80, and total cells recovered using Cell Dissociation Buffer for analysis by flow cytometry. The phagocytic index was calculated as follows: % Phagocytosis = 100 • (CellTracker^+^, F4/80^+^ macrophages/total macrophages).

### Microscopy

PBS-washed cells were re-suspended in cRPMI at 4 × 10^5^ cells/mL, incubated at 37 °C for 2 h with 125 ng/mL B6H12 or CC2C6, and fixed with BD CytoFix buffer, and labelled with Alexa Fluor488-conjugated secondary antibodies. All washes were with PBS. Labelled cells were re-suspended in Prolong Gold with DAPI (Invitrogen), mounted onto coverslips and allowed to cure overnight. Images were acquired on an Olympus IX81 microscope (20 × dry or 60 × oil objectives) equipped with a CoolSnap HQ2 camera (Photometrics) and controlled by Metamorph® software (Molecular Devices). Post-acquisition processing was performed on ImageJ.

### Hemagglutination assay

Red blood cells (RBC) were obtained from a healthy donor, washed and re-suspended as a 2% RBC solution in PBS. B6H12, BRIC-126, and CC2C6 antibodies at the indicated final concentrations was added to RBCs in round bottom 96-well microplates, incubated for 2 h at 37 °C, and the plate imaged on the Bio-Rad Gel Doc^TM^ XR + imaging system.

### CD47-internalization assay

PBS-washed cells were re-suspended at 5 × 10^5^ cells/mL in cRPMI, with or without 125 ng/mL CC2C6, B6H12 or 9F10 antibody treatments, and incubated for 2 h to 4 days (D0-D4) at 37 °C. At the indicated time points, untreated samples were removed for labeling of the corresponding cell surface receptors with primary and fluorescence-conjugated secondary antibodies, while treated samples received only secondary antibodies. Flow cytometry GMFI results for each sample was used to calculate % cell surface receptor expression as follows: 100*[(sample GMFI−background GMFI)/(maximum GMFI – background GMFI)]; where D0 samples were set at 100%.

## Results

### CC2C6-mAb induces cell death in a CD47-dependent manner

**Fig. 1:**
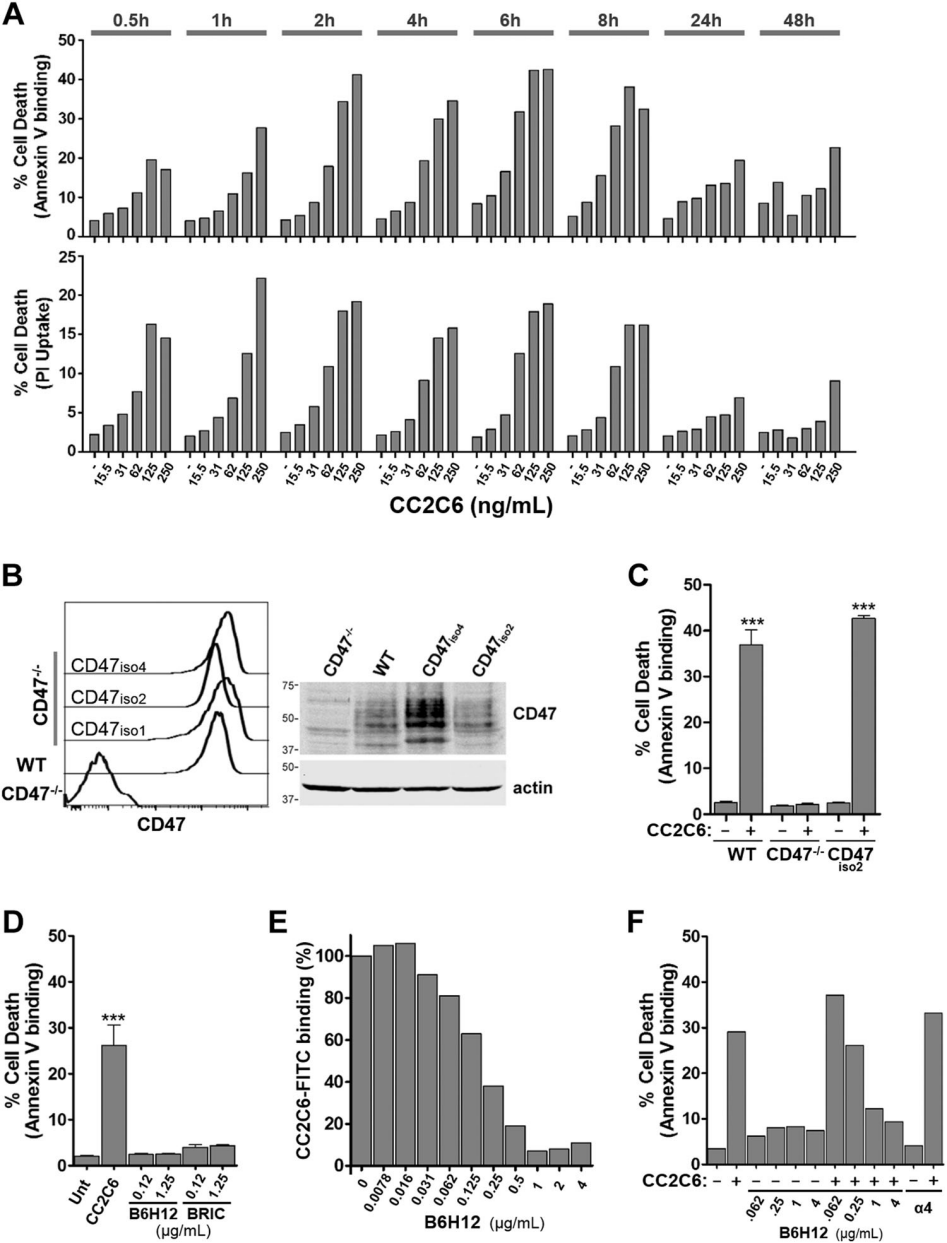
CC2C6 mAb induces cell death via CD47. **a** WT Jurkat cells were incubated with CC2C6 at the concentrations and durations as indicated, and cell death assayed by flow cytometry for Annexin V binding and PI uptake. Plotted is the percentage of dead relative to total cells. **b** CD47 expression in WT, CD47^−/−^, and CD47^−/−^ clones stably re-expressing CD47. Left panel: flow histograms. Right panel: western immunoblot. **c** WT, CD47^−/−^, or CD47^−/−^/CD47iso2 cells were incubated with 125 ng/mL CC2C6 for 2 h and the percentage of cell death determined using Annexin V binding. **d** Cell death comparison of WT cells treated with 125 ng/mL CC2C6, or with 125 ng/mL and 1.25 µg/mL B6H12 or BRIC-126 for 2 h. **e** WT cells were incubated with B6H12, washed, and then with CC2C6-FITC (as indicated in Methods and Materials). CC2C6-FITC binding was assessed by flow cytometry and set as 100% in the absence of B6H12. **f** WT cells were incubated with B6H12, washed, and then with 125 ng/mL CC2C6 for 2 h before determination of cell death by flow cytometry. As a control for B6H12, cells were also incubated with a mAb specific for α4-integrin. Error bars represent the standard deviation for *n* = 3 replicates, ***p < 0.001. **(c–f**) are representative of three independent experiments

CD47-ligation is known to induce Jurkat T-ALL cell death^14,15,21,22,28^. To evaluate if CC2C6-mAb induces cell death, we performed a time- and concentration-dependent experiment using the Annexin V-binding and PI-uptake assays. Jurkat cells treated with CC2C6 led to exposure of phosphatidylserine and membrane rupture as indicated by PI uptake within 30 min (Fig. 1a, Supp Fig. 1). The maximal fraction of cell death (≈40% by Annexin V assay) was achieved with 125-250 ng/mL CC2C6 for 2-8 h, while longer incubations of 24-48 h yielded lower percentages. For subsequent experiments, we routinely employed 125 ng/mL CC2C6 with 2 h incubation for short-term, or with 24-48 h incubation for long-term induction.

To assess if CC2C6-induced death is CD47-dependent, we used CRISPR-Cas9 to derive an isogenic CD47^−/−^ Jurkat cells. Sequencing of the targeted genomic loci showed that clone JC47-2-4 carries a homozygous frame shift mutation within exon 1 (Supp Fig. 2A), predicted to encode a truncated protein of 23 amino acids. We observed no morphological or growth defects in this or other independently isolated CD47^−/−^ clones (not shown); for brevity, JC47-2-4 was used as the representative CD47^−/−^ cells in all reported assays. To rescue CD47 function, we transfected JC47-2-4 cells to re-express CD47, isolated clonal populations and stable expressers by flow sorting, and confirmed their expression by flow and immunoblots (Fig. 1b). CC2C6 failed to induce CD47^−/−^ cell death, while CD47 re-expression restored this ability (Fig. 1c, Supp Fig. 2B). Since CD47 exists in alternately spliced isoforms encoding different C-terminal cytosolic tails with unknown function, we assessed, and found that CC2C6-induced comparable cell death in CD47^−/−^ cells expressing CD47-iso1 (no tail), iso2 (predominant isoform in leukocytes^16^) or iso4 (longest), suggesting the cytosolic tail plays an insignificant role (Supp Fig. 2B). CC2C6-induced death was also observed in JinB8 cells, an independently derived CD47-deficient cells, confirming the requirement of the CD47 receptor (Supp Fig. 2C).

We also found that CC2C6-induced death was comparable for cells incubated in serum-containing or serum-free media, suggesting that serum thrombospondin as a CD47-ligand contributes minimally to the death-inducing effects (Supp Fig. 3A). Next, we compared CC2C6 with other mAbs targeting CD47: the widely used B6H12^14,15,28^, and BRIC-126. As expected, B6H12 or BRIC-126 in solution induced minimal death, even at 10-fold higher concentrations compared to CC2C6 (Fig. 1d). CC2C6 and B6H12 likely shared an epitope on CD47 since cells pre-incubated with B6H12 effectively prevented binding of CC2C6, as well as CC2C6-mediated death (Fig. 1e,f). It’s been suggested that CD47-ligation may lead to receptor internalization, thus we compared cell surface levels of CD47 and ITGA4 (an abundant integrin in Jurkat) following ligation with antibodies. Our results indicated that CC2C6-ligated CD47 internalized at the same rate as ITGA4 over 4 days, while internalization of B6H12-ligated CD47 within the first 24 h is less (Supp Fig. 3B). Furthermore, we found that cells re-stimulated with a fresh bolus of CC2C6 following the initial 24 h treatment did exhibit increased death (Supp Fig. 3C), suggesting either loss of CC2C6 or attenuation of CD47 activity over time that can be overcome with further CC2C6-dosing. In sum, our results indicate that CC2C6-induced cell death is CD47-dependent, but unlike B6H12, occurs efficiently in soluble phase without immobilization.

### CC2C6-treatment promotes phagocytosis, hemagglutination and cell aggregation

**Fig. 2:**
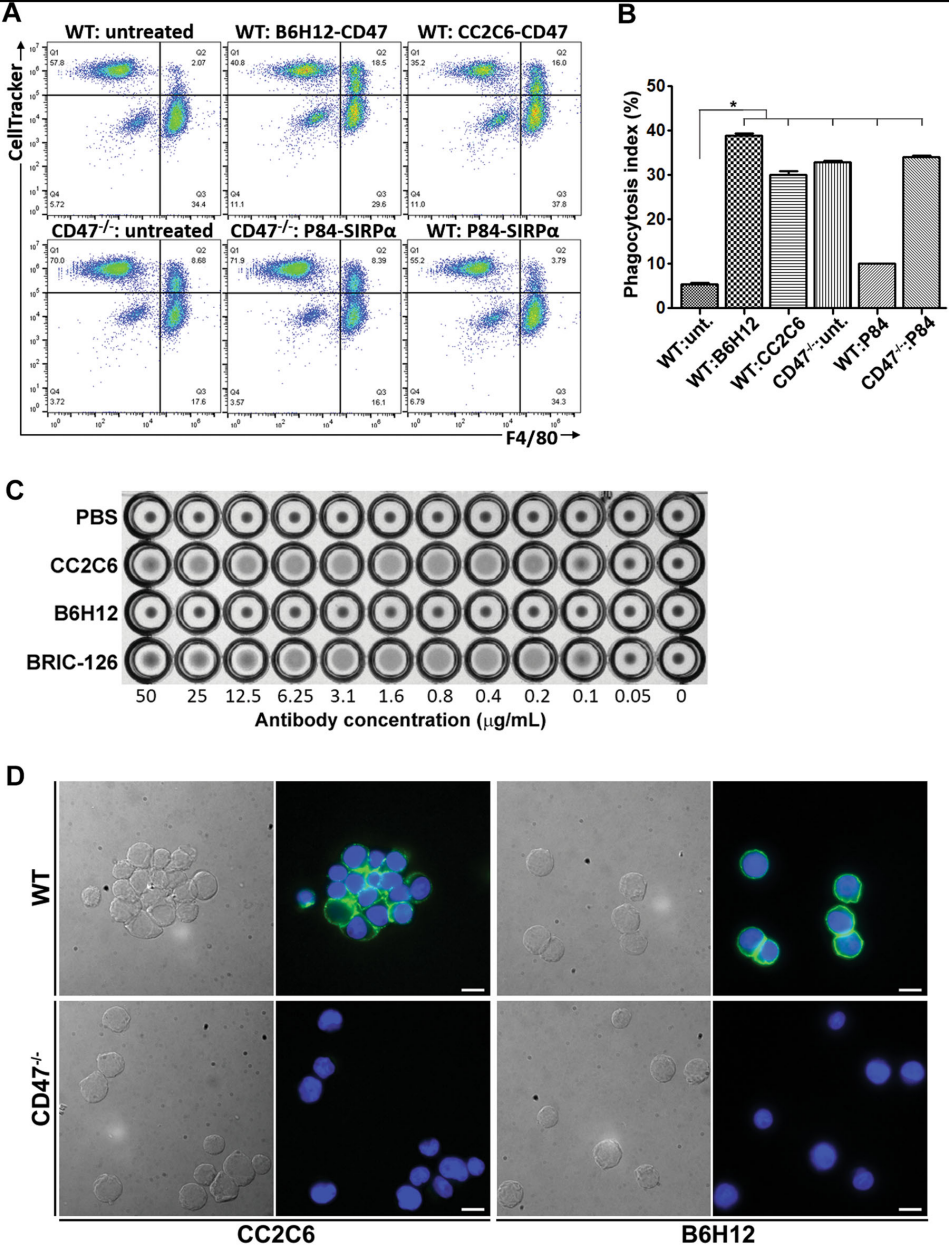
CC2C6 inhibits anti-phagocytic signal of CD47. **a** Cell Tracker-labelled WT or CD47^-/-^Jurkat cells were untreated or treated with B6H12 or CC2C6 prior to their incubation with primary mouse macrophages (F4/80 labeled) for 2 h. In two samples, macrophages were treated with the SIRPα-neutralizing antibody, P84, prior and during incubation with WT or CD47^−/−^ cells. Shown is the flow cytometry plots of the mixed population, where double positive cells indicate Jurkat cells phagocytosed by macrophages. **b** The phagocytic index for the data shown in (**a**) was calculated as follows: 100 × (CellTracker and F4/80 double + ve cells) / total macrophages. Error bars represent the standard deviation for *n* = 3 replicates; *p < 0.01 relative to untreated WT. Representative of two independent experiments. **c** Hemagglutination assay of human erythrocytes incubated with increasing concentrations of the CD47-antibodies, CC2C6, B6H12 or BRIC-126. **d** DIC and immunofluorescence imaging of WT or CD47^−/−^ cells treated with CC2C6 or B6H12 and mounted in suspension. Green: CD47; Blue: DAPI. Bar: 10 μM

B6H12 can effectively neutralize CD47’s anti-phagocytic signal^6,25^, thus we determined if CC2C6 possesses this function-blocking activity. WT cells treated with CC2C6 or B6H12 effectively promoted their phagocytic uptake by murine macrophages when compared to non-treated cells (Fig. 2a,b). The phagocytic index of WT cells treated with either antibody is comparable to non-treated CD47^−/−^ cells, suggesting efficient neutralization of CD47’s anti-phagocytic signal. Rather surprisingly, our efforts to neutralize macrophage SIRPα with the P84 antibody yielded a significant, albeit modest, increase in phagocytosis of WT cells.

Certain CD47-antibodies have been reported to promote blood hemagglutination and homotypic cell-cell interaction. We assessed, and found that CC2C6 concentrations typically used in our experiments induced hemagglutination of human erythrocytes comparable to BRIC-126, but not B6H12^29^ (Fig. 2c). WT cells treated with CC2C6 resulted in considerable cell aggregation when compared to B6H12, or to CD47^−/−^ cells treated with either antibody (Fig. 2d). Immunofluorescence imaging also revealed an increase in punctate CD47-clustering at cell-cell interfaces with CC2C6-treatment when compared to the more diffuse staining exhibited by B6H12.

Therefore, unlike other commercially available α-CD47 antibodies, we found that CC2C6-mAb in solution efficiently induced Jurkat T-ALL cell death in a CD47-dependent manner. In addition, CC2C6-mAb retained the CD47-neutralizing activity that promotes phagocytic uptake of lymphoblasts by macrophages. Taken together, the dual anti-tumour activities of CC2C6 has the potential to maximize the therapeutic benefits for targeting CD47.

### CC2C6 synergizes with chemotherapeutic agents to induce cell death

**Fig. 3:**
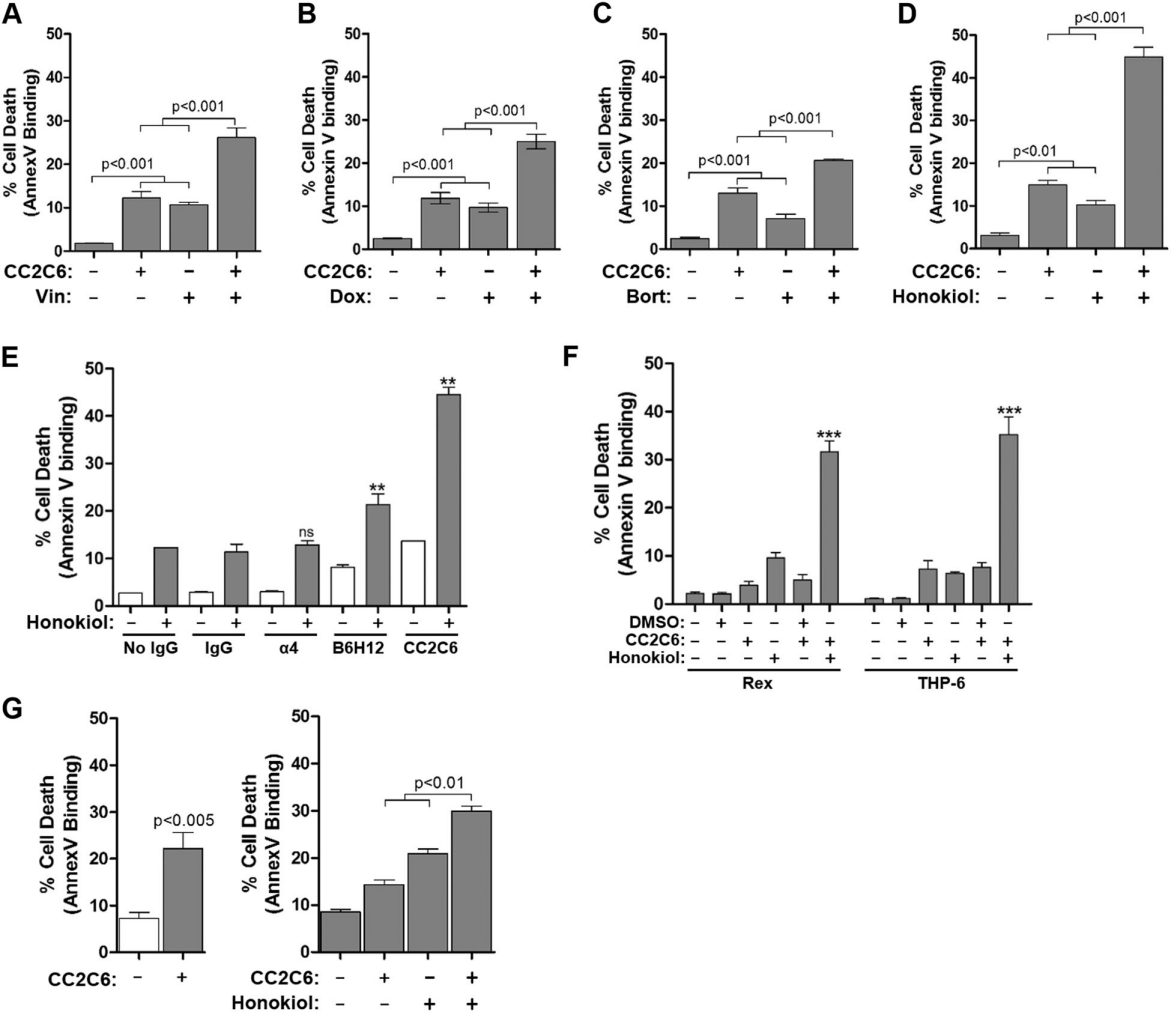
CC2C6 combines with low dose cytotoxic agents to induce cell death. Jurkat cells were incubated with or without 125 ng/mL CC2C6 alone or in combination with (**a)** 0.8 ng/mL vincristine, **(b)** 7.5 ng/mL Doxorubicin, or **(c)** 2 ng/mL bortezomib for 48 h, or with (**d**) 30 μM Honokiol for 24 h, and cell death assayed by flow cytometry for Annexin V binding. **e** As in (**d)**, Honokiol was used in combination with anti-CD47 antibodies CC2C6 or B6H12, an isotype control IgG, an anti-α4-integrin antibody, or without antibodies. **f** As in (**d**), using the T-ALL cell lines Rex and THP-6. **g** Cell death assays on T-ALL blasts from a patient-derived xenograft (BD67-R2). Left panel, cells were treated with or without CC2C6 for 2 h. Right panel, cells were treated with or without CC2C6, honokiol, or both, for 24 h (as in **d**). Error bars represent the standard deviation for *n* = 3 replicates. ***p < 0.001; **p < 0.01. Representative of at least three independent experiments for (**a**–**d**, **f**) and of two independent experiments for (**e**, **g**)

Given reports that CD47-ligation induced cell death in a caspase-independent manner, we reasoned its effects may be complementary to cytotoxic agents that induce caspase-dependent apoptosis. We evaluated the chemotherapeutic agents doxorubicin, vincristine, and the proteasome inhibitor, bortezomib^4,30^ by treating cells with a sub-optimal drug concentration (based on kill curves, Supp Fig. 4) with or without CC2C6 for 48 h. CC2C6 alone, or any one of doxorubicin, vincristine or bortezomib, induced only low to moderate levels of cell death (Fig. 3a–c). This level increased in an additive manner upon combining CC2C6 with drug-treatment, suggesting that CC2C6 may have potential therapeutic benefits for reducing cytotoxic drug dosing while maintaining the ability to induce tumour cell death.

The cytotoxic effects of agents evaluated thus far required at least 48 h of treatment, while optimal CC2C6-induction of cell death occurs within 2–24 h (Fig. 1a). Therefore, we evaluated additional compounds to identify a pairing that better complements the effects of CC2C6. We found that honokiol, a small molecule isolated from *Magnolia officinalis* with known anti-tumour properties^31–33^, induces significant levels of cell death within 24 h (Supp Fig. 4A). The combinatorial effects of CC2C6 and honokiol treatment for 24 h were synergistic, and not simply additive (Fig. 3d). To assess if CD47-expression mitigated the sensitivity of cells to the cytotoxic agents, we conducted kill curve assays for WT and CD47^–/–^ cells, and found that loss of CD47 did not impact significantly upon their individual susceptibilities to honokiol, doxorubicin or vincristine (Supp Fig. 4).

To confirm if the synergistic effects mediated by honokiol and CC2C6 was specific for CD47, we conducted additional antibody controls. Co-incubation of cells with non-specific IgGs, or with an antibody targeting ITGA4, did not change cell death levels induced with or without honokiol (Fig. 3e). We noted that B6H12-treatment for 24 h induced a small but significant increase in cell death, which further combined with honokiol in an additive manner (Fig. 3e). CC2C6 and honokiol also combined synergistically to achieve the highest cell death levels which exceeded all other tested conditions (Fig. 3e). This suggests that B6H12 in soluble form can promote CD47-mediated cell death, albeit at reduced efficiency or kinetics compared to CC2C6. Importantly, CC2C6 can synergize with other agents and identifies honokiol as a particularly effective agent when used in combination.

To evaluate the general effectiveness of CD47-ligation induced death, we treated additional T-ALL cell lines with CC2C6. In contrast to Jurkat cells, CC2C6 as a mono-agent was not as effective at 2 or 24 h of treatment in the cell lines tested (Supp Fig. 5). All cell lines expressed abundant surface levels of CD47 (not shown), thus it remains unclear what mechanisms constitute the increased activity for CD47-ligation induced death in some cells but not others. Since CC2C6 and honokiol combined synergistically in Jurkat cells, we repeated the combination treatment for the seemingly non-responsive cells. Two of these, Rex and THP-6, were significantly responsive to the combined treatments (Fig. 3f, Supp Fig.  5). CC2C6 also induced cell death in a patient-derived T-ALL; 2 h treatment with CC2C6 alone, or 24 h treatment with CC2C6 and honokiol combined to induce significant death compared to the non-treated controls (Fig. 3g). Together, these results indicate that CC2C6 is a unique mAb useful for targeting CD47 on T-lymphoblasts to induce cell death, either as a mono-agent or as a sensitizing agent in combination with conventional chemotherapeutics.

### Characterization of CC2C6-induced cell death

**Fig. 4:**
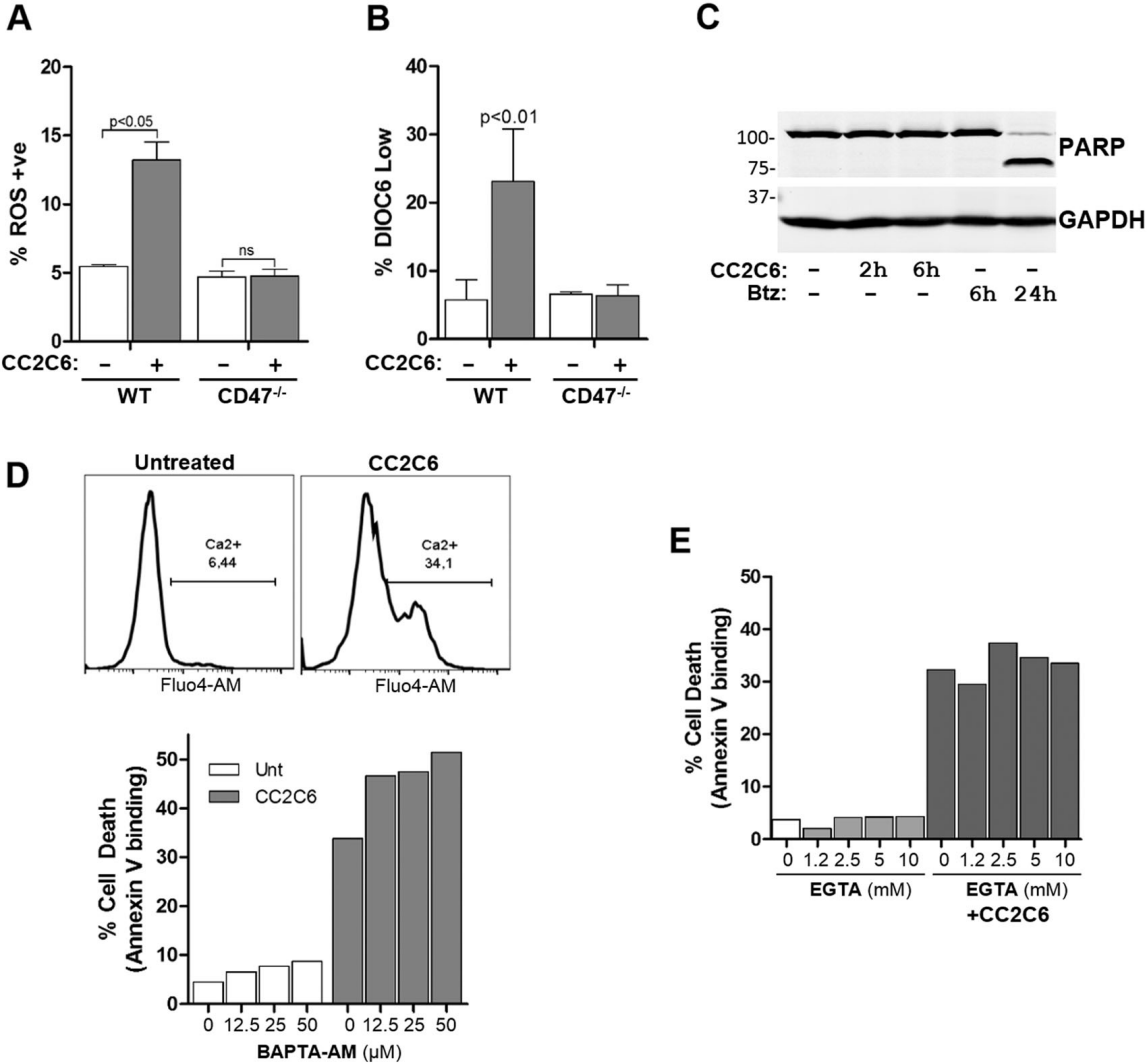
Characterization of CC2C6-induced cell death. **a** WT and CD47^−/−^ Jurkat cells were treated with or without 125 ng/mL CC2C6 for 2 h and reactive oxygen species (ROS) generation assessed by flow cytometry using MitoSox labeling. **b** As in (**a**), loss of mitochondrial membrane potential (MMP) was assessed by flow cytometry using DiOC_6_ labeling. **c** Jurkat cells were untreated or treated with 125 ng/mL CC2C6 or bortezomib for the indicated times and cell lysates immunoblotted for PARP and GAPDH. **d** Top panel: Jurkat cells labelled with Fluo4-AM were treated with and without 125 ng/mL CC2C6 for 2 h and intracellular Ca^2+^ assessed by flow cytometry. Bottom panel: Jurkat cells were untreated or pretreated with BAPTA-AM at the indicated concentrations, followed by treatment with and without 125 ng/mL CC2C6 for 2 h. Cell death was assayed by flow cytometry using Annexin V binding. **e** As in (**d**), Jurkat cells were incubated with and without 125 ng/mL CC2C6 and EGTA at the indicated concentrations, and cell death assessed by flow cytometry. Error bars represent the standard deviation for *n* = 3 replicates. Representative of three independent experiments

It was shown previously that CD47-ligation is coupled to increased ROS, decreased mitochondrial membrane potential (MMP), and proceeded via a caspase-independent pathway^15,21,34^. As this is the first characterization of CC2C6-induced cell death, we investigated involvement of these pathways. Indeed, CC2C6-treatment stimulated increased mitochondrial ROS and decreased MMP in a CD47-dependent manner (Fig. 4a,b). CC2C6-treatment did not produce measurable caspase activation, as reported by PARP-1 cleavage assays (Fig. 4c), or with a luciferase-based caspase substrate kit (Supp Fig.  6).

CD47-ligation is also known to stimulate increases in intracellular calcium^35–37^. It was reported that the 4N1K-related peptide induced cell death in a manner dependent on intracellular calcium elevation^38^. We found that CC2C6-treated Jurkat cells exhibited elevated Ca^2+^ levels, however, chelation of intracellular Ca^2+^ with BAPTA-AM, or of extracellular Ca^2+^ with EGTA, failed to diminish CC2C6-mediated cell death (Fig. 4d, e). In sum, our results are consistent with prior studies which show that CD47-ligation induced cell death via a caspase-independent pathway, concomitant with increased Ca^2+^ accumulation, ROS-generation and a collapse of the MMP.

### CC2C6-mediated cell death is modulated by Bcl-2 proteins

**Fig. 5:**
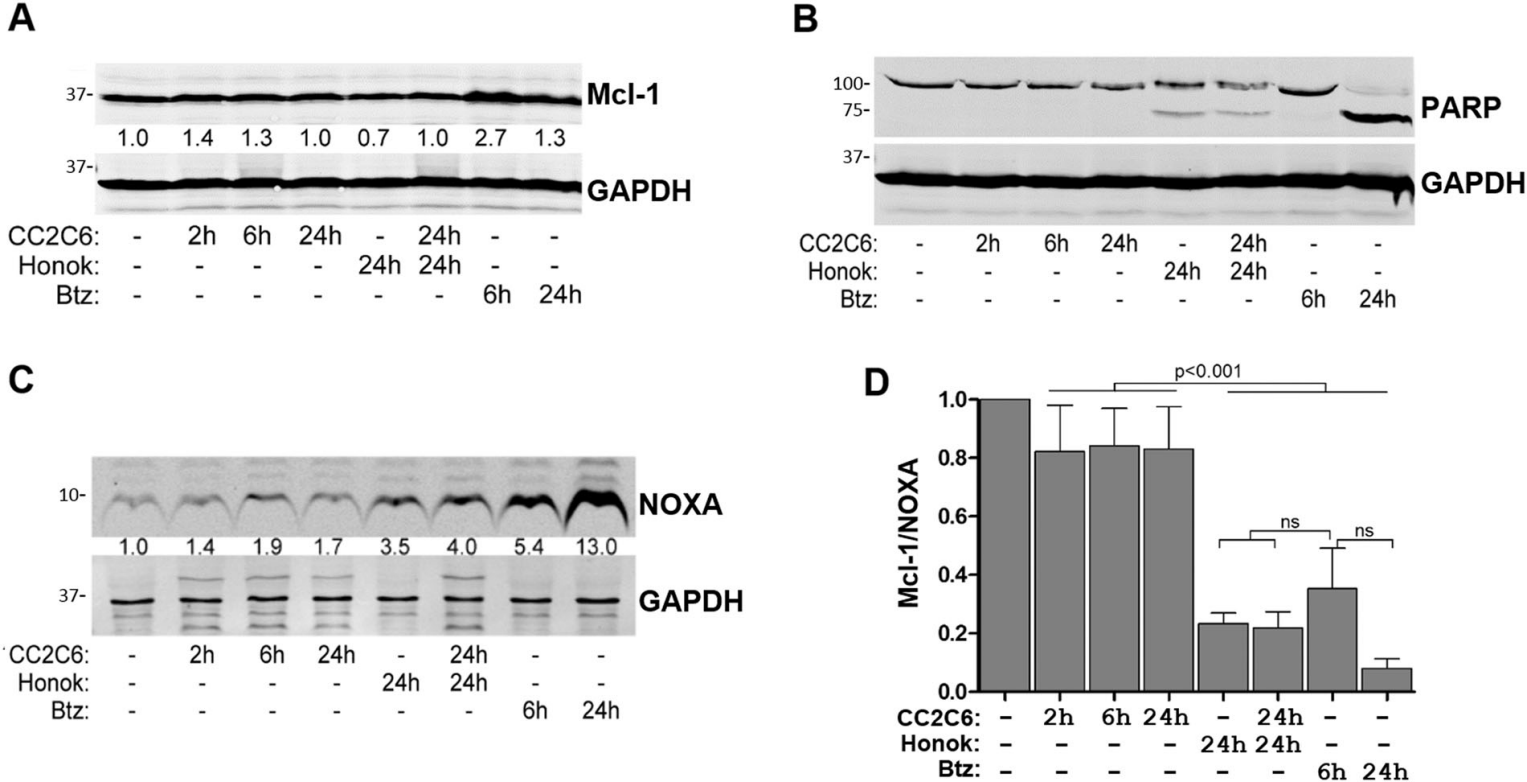
CC2C6-induced cell death is modulated by Mcl-1 and NOXA. Jurkat cells were treated with 125 ng/mL CC2C6, 40 μM Honokiol, or a combination thereof, for the indicated times. Cells were treated with 30 nM bortezomib as controls. Cell lysates were fractionated by SDS-PAGE and immunoblotted with antibodies to detect (**a**) Mcl-1, (**b**) PARP-1, and (**c**) NOXA. Fractions indicated under the bands represent the fold enrichment of Mcl-1 (**a**) and NOXA (**c**) relative to the untreated condition. GAPDH levels were used to compare protein loading and normalize expression for all lanes. Shown are representative blots from at least three independent experiments. **d** Calculation of Mcl-1:NOXA ratios for the indicated treatments using data from at least three independent experiments

To understand cell death induced by CD47-ligation, we evaluated the expression of key regulatory proteins involved in intrinsic apoptosis. Levels of the Bcl-2 family protein, Mcl-1, is typically downregulated upon apoptosis induction^39^. However, we consistently observed that CC2C6-treatment of Jurkat for 2 or 6 h, corresponding to conditions that induced high death rates (Fig. 1a), have increased Mcl-1 levels compared to untreated cells, or to 24 h-treated cells (Fig. 5a). Consistent with previous findings showing honokiol decreases Mcl-1 in tumour cells^40^, we found that cells treated with honokiol for 24 h as a mono-agent have significantly reduced Mcl-1 expression (Fig. 5a). In comparison, cells co-treated with honokiol and CC2C6 for 24 h, conditions that induced high death rates (Fig. 3d), have increased Mcl-1 levels (Fig. 5a). Thus, unlike conventional chemotherapeutics which decrease Mcl-1 levels, the death-inducing effects of CC2C6 is affiliated with increased or stabilized Mcl-1 levels. We noted that PARP-1 cleavage products is not further elevated with the combined honokiol and CC2C6 treatment, when compared to honokiol alone (Fig. 5b), suggesting the synergistic effects of CD47-ligation on cell death remained caspase-independent.

Since Mcl-1 activity is selectively antagonized by the BH3-only and pro-apoptotic protein, NOXA^39^, we assessed if NOXA levels is modulated by CD47-ligation. Surprisingly, CC2C6-treatment resulted in increased NOXA levels, although this increase was moderate compared to honokiol or bortezomib treatments (Fig. 5c). Since CC2C6-treatment increased both Mcl-1 and NOXA, we analyzed their relative expression ratios to gain further context. Agents which induce caspase-dependent apoptosis, such as honokiol and bortezomib, both resulted in very low ratios of Mcl-1:NOXA proteins (Fig. 5d). Conversely, CC2C6-only treatment resulted in a modest decrease in this ratio, likely insufficient to initiate mitochondrial cytochrome c release and subsequent caspase activation. In combination, the high NOXA levels induced by honokiol greatly exceeds the modest increase in Mcl-1 mediated with CC2C6, thus honokiol can be seen as sensitizing cells to CD47-ligation induced cell death.

**Fig. 6:**
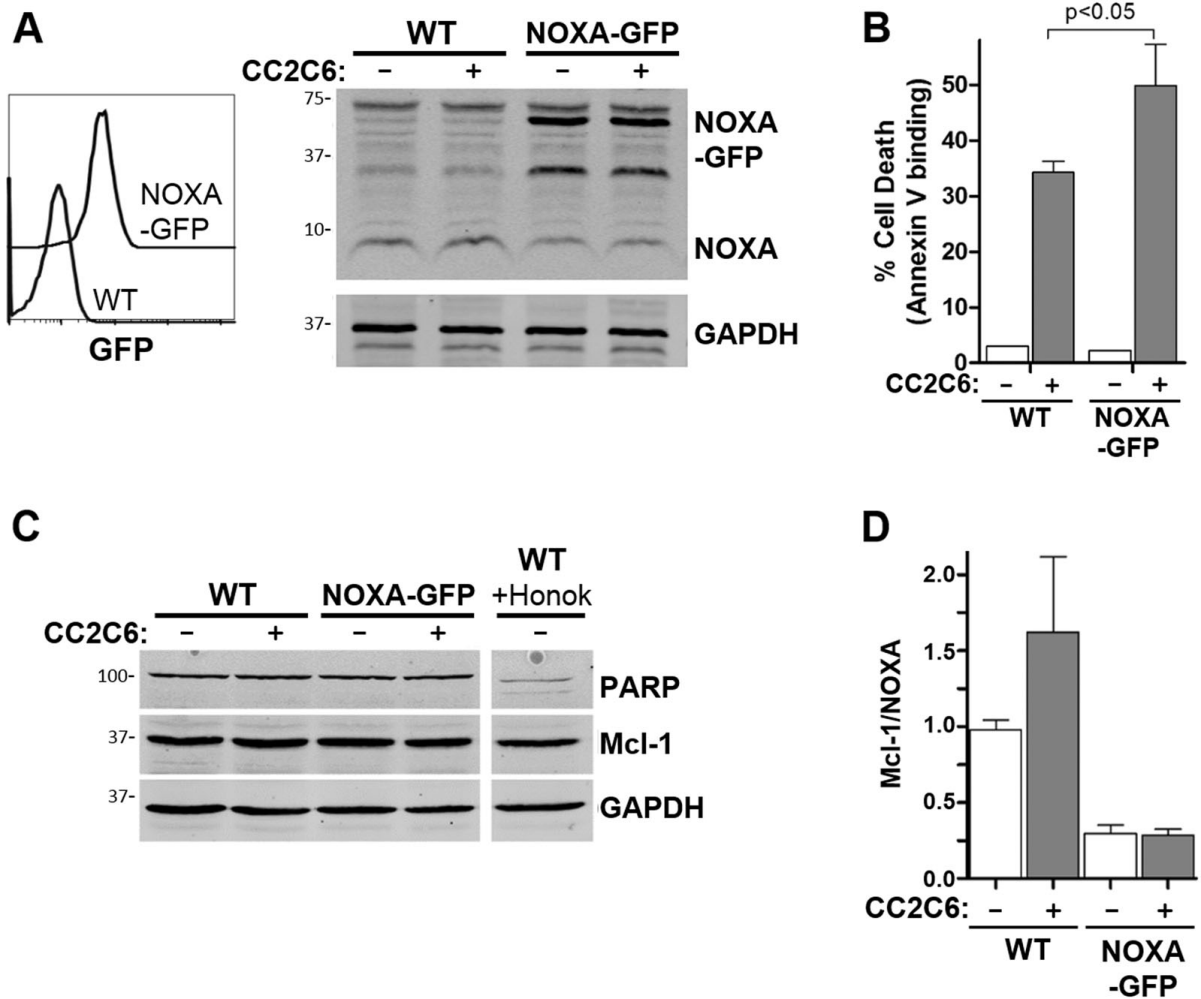
NOXA overexpression increases CC2C6-induced cell death. **a** Jurkat cells were transfected to express NOXA-GFP. Left panel, flow cytometry histograms of untransfected cells (WT) and a clonal derivative stably expressing NOXA-GFP. Right panel, immunoblot of cell lysates showing expression of NOXA, NOXA-GFP and GAPDH. **b** As indicated, cells were treated with and without 125 ng/mL CC2C6 for 2 h and cell death assessed by flow cytometry using Annexin V binding. Error bars represent the standard deviation for *n* = 3 replicates. **c** In addition, cell lysates were immunoblotted to assess PARP-1, Mcl-1 and GAPDH levels. Honokiol treated WT cells is included as positive control to show PARP-1 cleavage. **d** Calculation of Mcl-1:NOXA ratios for the indicated treatment from the immunoblots represented in (**a**, **c**) using data from at least three independent experiments

To determine if increased NOXA levels can sensitize cells to CD47-ligation induced death, we derived Jurkat cells transfected to stably express GFP-tagged NOXA (Fig. 6a). Whereas overexpression of NOXA did not affect basal levels of cell death, CC2C6-treatment of NOXA-GFP cells resulted in increased death when compared to the parental controls (Fig. 6b). Again, cleavage of PARP-1 was not observed for CC2C6-treated NOXA-GFP cells (Fig. 6c), indicating that caspases remained inactivated. The Mcl-1:NOXA ratios of NOXA-GFP cells were significantly lower than the WT counterpart, with or without CC2C6-treatment (Fig. 6d). In sum, our results suggest that cell death mediated by CD47-ligation with CC2C6 involves upregulation of the Bcl-2 proteins, Mcl-1 and NOXA, in a manner that does not disrupt their ratiometric balance sufficiently to induce caspase-dependent cell death.

## Discussion

CD47 is a tumour cell surface antigen that is currently the target of several clinical trials utilizing biologics aimed at neutralizing its function as an anti-phagocytic receptor. However, its potential as an inducer of cell death remains largely unexploited. Here we describe the direct induction of cell death with a commercially available anti-CD47 antibody, CC2C6, originally described for its ability to efficiently block binding of soluble SIRPα to CD47-expressing cells^41^. We report that CC2C6 in soluble form effectively induces T-leukemic cell death in a CD47-dependent manner. The CC2C6-induced phenomena are similar to those previously reported for other CD47-antibodies, including exposure of phosphatidylserine, increased Ca^2+^_ί_, loss of mitochondrial membrane potential, ROS generation, and cell death independent of caspase activation^14,15,21,22,28^. Further to its cell death-inducing properties, CC2C6 also functionally blocked the epitope responsible for the anti-phagocytic activity of CD47 in a manner comparable to B6H12. This dual anti-tumour activity enhances the utility of CC2C6 as a single biologic therapeutic for targeting CD47-expressing tumours.

Several phase I/II trials using the humanized CD47-antibody Hu5F9-G4 for several malignancies, either alone or in combination with chemotherapeutics (NCT02216409, NCT02678338, NCT02953509, NCT03248479, NCT02953782) are ongoing^42^. Trials in non-human primates indicated that the antibody was well tolerated, though it induced minor anemia that resolved within weeks^12^. In a human Phase I trial, all 16 participants suffered reversible G1 or G2 anemia associated with the priming dose^43^. Indeed, a limitation of CD47-antibodies as a clinically viable therapeutic is the propensity for inducing erythrocyte hemagglutination^29,44,45^. In that regard, CC2C6 in its current form would require modification to reduce its hemagglutination and other blood cell aggregation potential. For example, a single-chain antibody fragment with a short linker completely removed the hemagglutination effects of the MABL-antibody^45^. Ideally, any modification of CC2C6 should retain its ability to induce tumour cell death and promote their phagocytic uptake by professional phagocytes.

A desired CC2C6 property is the ability to efficiently induce cell death in soluble form, in contrast to B6H12 and BRIC-126, which require immobilization^21,22^. Other CD47-antibodies able to induce death in solution have been reported, although these remain non-commercially available. The first report of CD47-ligation induced death was with the mAb Ad22 or 1F7^14^. Similar to CC2C6, these antibodies shared overlapping epitopes with B6H12, and induced death in solution with comparable kinetics. Later, the F(ab’)_2_ fragment of MABL was shown to be sufficient for cell death induction in vivo^46^. However, both the intact mAb and its F(ab’)_2_ fragment caused hemagglutination, which precipitated development of a dimerized single chain variant that induced death without hemagglutination^17^. Wiersma et al. described a chimeric Ab that fused the CD47-binding fragment of B6H12 to the pro-apoptotic fragment of TRAIL, combining the anti-phagocytosis-blocking ability of B6H12 with TRAIL’s apoptosis-inducing effects^47^. Importantly, normal peripheral blood mononuclear cells were not sensitive to the anti-CD47:TRAIL mAb treatment.

Besides antibodies, thrombospondin (TSP) is the only natural ligand for CD47 shown to induce apoptosis of breast cancer cells^48^. The TSP-derivative C-terminal peptides, 4N1K and 7N3, were similarly shown to induce cell death in a number of cell lines in solution^38,49,50^. However, questions arose concerning the specificity of these peptides since 4N1K appears to bind cells and stimulate activities in a CD47-independent manner^51,52^. As the only commercially available CD47-antibody able to induce cell death in solution, CC2C6 should enable further studies that enhance understanding of a 'don’t eat me' tumour cell antigen that can be exploited to turn on itself.

We observed that short-term incubation of cells with CC2C6 (≤8 h) induced a greater percentage of cell death compared to long-term incubations (≥24 h). Several possibilities may account for this phenomenon. The CD47-receptor may be internalized following ligation, hence decreasing continued induction of cell death. Our data indicated CD47-internalization following CC2C6-treatment occurred at a rate greater than that induced by B6H12, but no greater than an integrin receptor used for comparison. Total surface expression remained comparable, suggesting adequate surface replenishment of receptors, hence receptor expression was deemed not likely to be a limiting factor. This favours an explanation whereby CC2C6-CD47 binding for extended periods may desensitize CD47-signaling. Likewise, CC2C6 may be unstable in solution resulting in loss of activity. It has been observed that some cells, including Jurkats, shed CD47-containing extracellular vesicles^53,54^, potentially reducing the number of antibody-bound cell surface CD47 and decreased induction of cell death. We acknowledge that the reduced percentage of cell death following long-term incubation with CC2C6 can be rationalized by continued growth and division of viable cells. This suggests that a certain fraction of cells is refractory to CD47-ligation induced death and remains so even with excess CC2C6. To address this, we re-stimulated cells following 24 h incubation with a fresh bolus of CC2C6, and observed death rates approaching that of 2 h only induction (Supp Fig. 3C). Further investigation will be required to resolve complex stability and the loss of activity seen with prolonged CC2C6-incubation.

Despite the reduced effects of CC2C6 after 24 h incubation, we show that CC2C6 remained effective in combination therapy, even when incubated for as long as 48 h. Indeed, combination treatment using low toxicity concentrations of three different cytotoxic agents used in therapy of leukemias, vincristine, doxorubicin, or bortezomib, all had additive effects when used with CC2C6 (Fig. 3). The combinations likely complemented one another since these agents induce death via caspase-activation^23^, whereas CD47-mediated death employs a caspase-independent pathway. Importantly, the cytotoxic effects for each agent in isolation is minimal, raising the added benefit of reduced dosing while maintaining the anti-tumour therapeutic benefits.

Honokiol is one of two bioactive constituents of the medicinal herb *Magnolia officinalis* with known potent anti-tumour effects that induce cell death via caspase activation^40,55–58^. This small molecule bi-phenolic lignan was shown to target NF-κB,  STAT3, EGFR and m-TOR pathways, with low cytotoxicities in animal models and therefore considered clinically safe^57^. We evaluated honokiol in combination with CC2C6 since honokiol induces significant apoptosis in 24 h, as opposed to 48 h required for doxorubicin or vincristine. This reduced treatment time may have contributed to the synergistic effects observed with honokiol and CC2C6 at 24 h, compared to mainly additive effects seen for the other combinations at 48 h. Unexpectedly, we found that honokiol combined with B6H12 in solution to achieve a small but significant additive effects on cell death. It remains unclear why B6H12 in solution exhibit slower kinetics compared to soluble CC2C6, however, having two different monoclonal antibodies able to induce cell death in a CD47-dependent manner affirms the strategy of targeting CD47 as an anti-tumour therapy.

CC2C6, as a commercially available CD47-antibody, presented a unique opportunity to further characterize cell death pathways induced upon CD47-ligation. We found that CC2C6-treatment induced detectable increases in Mcl-1 and NOXA, with the levels of both maintained at a somewhat constant ratio. This observation is consistent with CD47-induced death occurring in a caspase-independent manner, since mitochondrial permeabilization, cytochrome *c* release, and ultimately, caspase activation, requires a significant decrease in the Mcl-1:NOXA ratio to occur. Indeed, co-treatment of cells with CC2C6 and a caspase-activating agent, such as bortezomib or honokiol, altered this ratio in favour of the pro-apoptotic NOXA. We also found that Jurkat cells expressing NOXA-GFP had significantly increased CC2C6-induced death without impacting basal apoptotic levels, consistent with NOXA’s role as the main regulator of Mcl-1 and sensitizer of the intrinsic apoptotic pathway^39,59,60^, and likely as a consequence of decreased Mcl-1:NOXA ratio. To our knowledge, this is the first report implicating Bcl-2 family proteins as key signalling events in CD47-ligation induced cell death. Our data is consistent with a model in which CC2C6-CD47 ligation up-regulates both Mcl-1 and NOXA levels without affecting the overall balance of these proteins, and is therefore insufficient to promote caspase activation and mitochondria pore formation. Importantly, we show that CC2C6-mediated cell death can synergize with existing and novel chemotherapies, perhaps allowing the reduction of toxic reagents that are known to have serious, long-term sequelae, without compromising their tumouricidal effects.

## Electronic supplementary material

Supplemental Figures

Supplementary figure legends
