# Supplementary figures and images for: CD47-ligation induced cell death in T-acute lymphoblastic leukemia
Source: Cell Death Dis. 2018 May 10;9(5):544. doi: 10.1038/s41419-018-0601-2 (PMC5945676; doi:10.1038/s41419-018-0601-2)

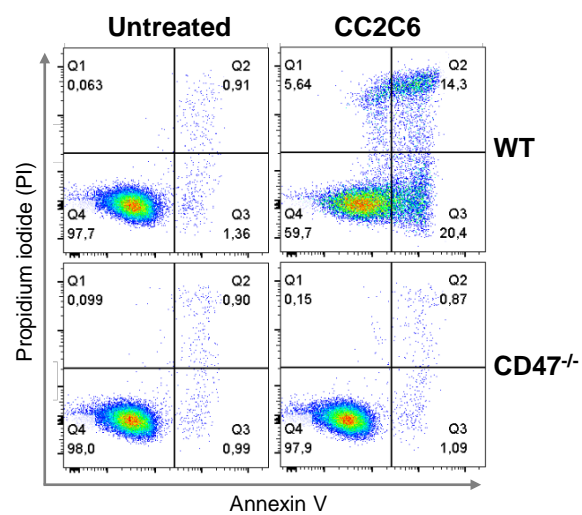

**Supp Figure 1**

**A**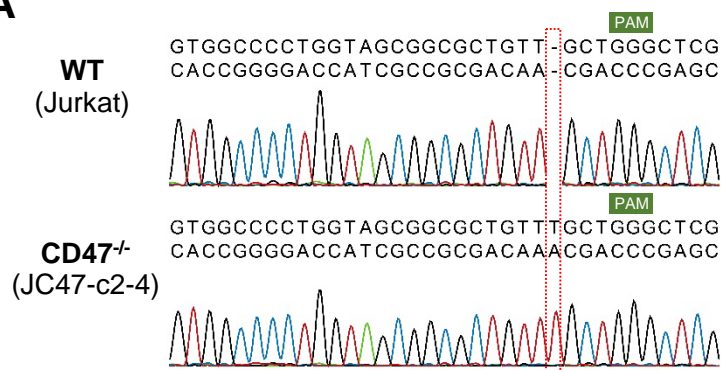**B**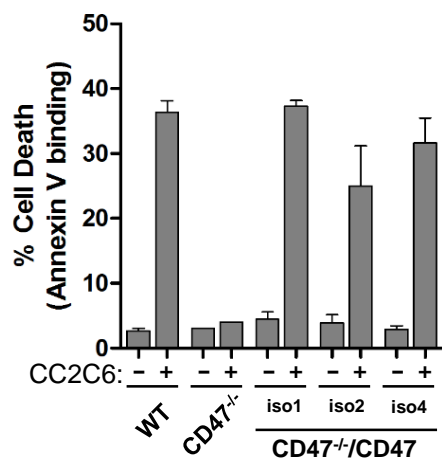**C**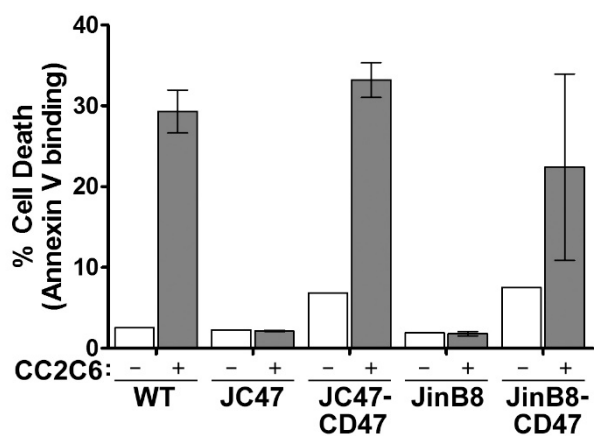**Supp Figure 2**

**A**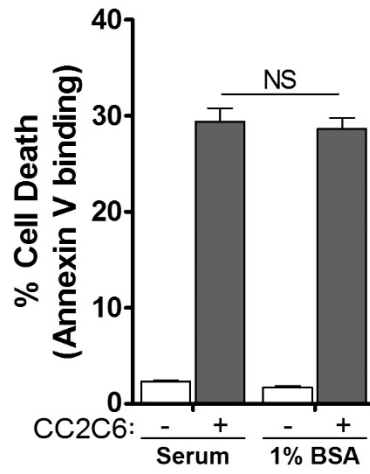**B**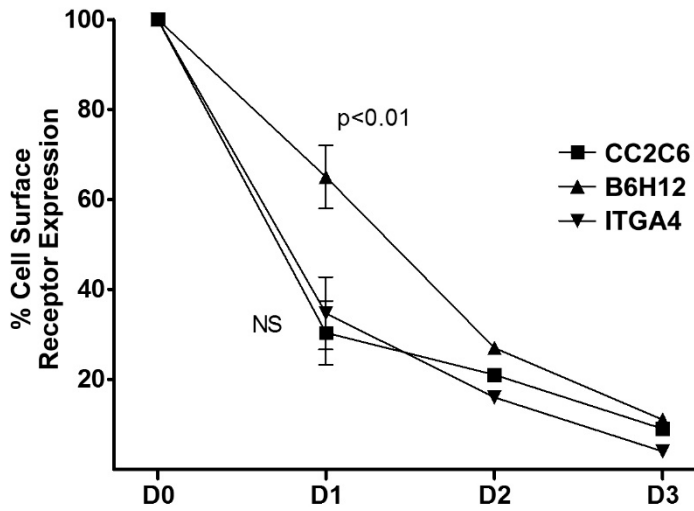**C**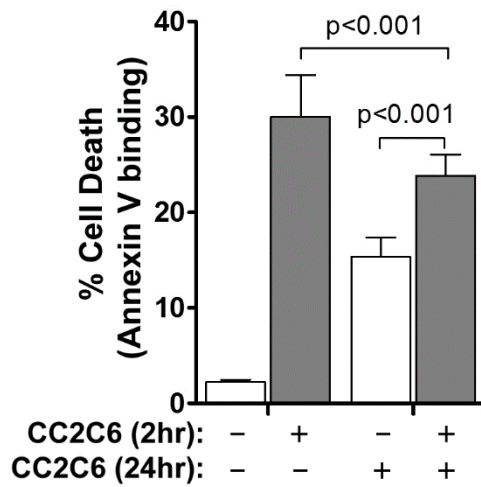**Supp Figure 3**

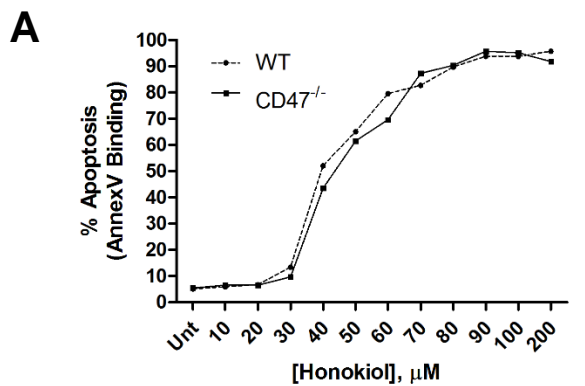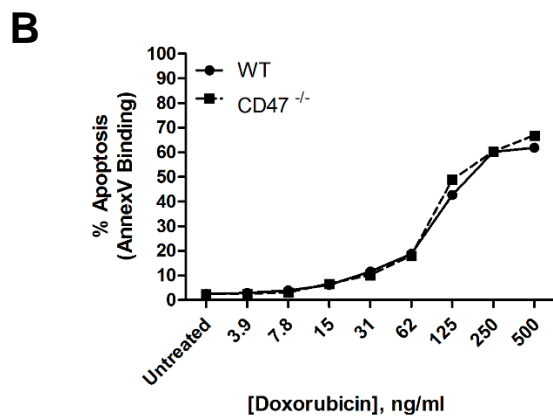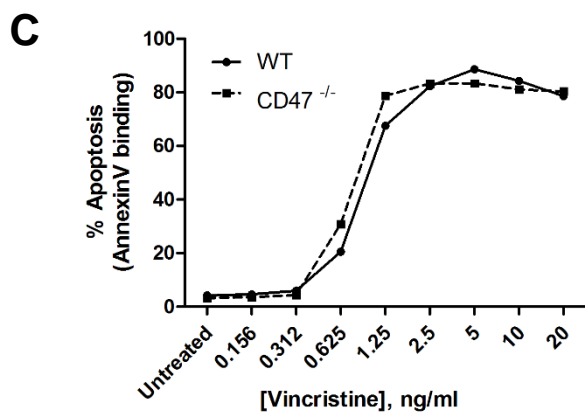

**Supp Figure 4**

**A**

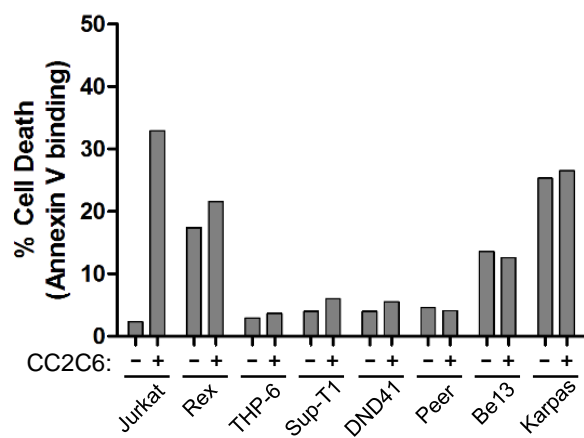

**B**

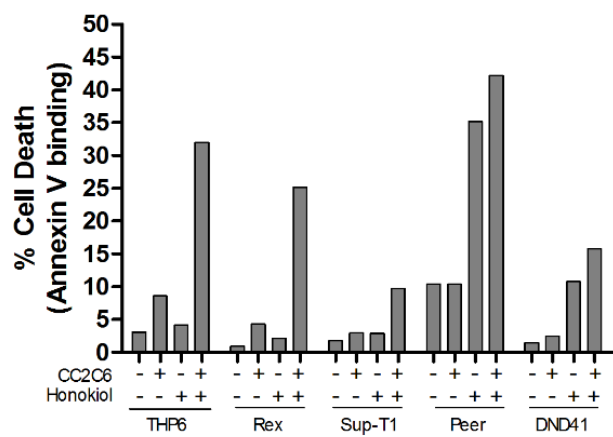

**Supp Figure 5**

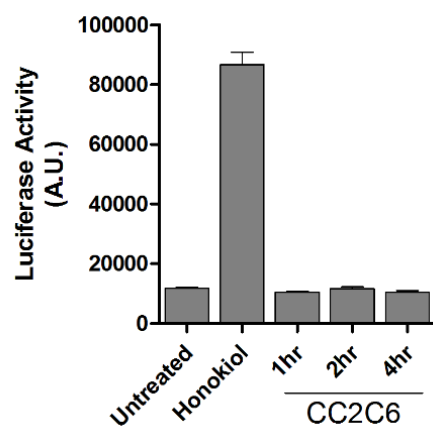

**Supp Figure 6**

Supplement: Supplementary file 1 — Supplemental Figures [file 41419_2018_601_MOESM1_ESM.pdf]
