# Supplementary material for: CD47-ligation induced cell death in T-acute lymphoblastic leukemia
Source: Cell Death Dis. 2018 May 10;9(5):544. doi: 10.1038/s41419-018-0601-2 (PMC5945676; doi:10.1038/s41419-018-0601-2)
Supplement: Supplementary file 2 — Supplementary figure legends [file 41419_2018_601_MOESM2_ESM.docx]

**CD47-ligation induced cell death in T-Acute Lymphoblastic Leukemia**

Pascal Leclair^1^, Chi-Chao Liu^1^, Mahdis Monajemi^2^, Gregor S. Reid^1,3^, Laura M. Sly^1^, Chinten James Lim*^1,3^

Depts. of Pediatrics^1^, Medicine^2^, and the BC Children’s Hospital Research Institute, ^3^Michael Cuccione Childhood Cancer Research Program, B.C. Children’s Hospital and University of British Columbia, Vancouver, British Columbia, Canada V5Z 4H4

*Corresponding author: Chinten James Lim, Department of Pediatrics, University of British Columbia, 3092-950 West 28^th^ Ave., Vancouver, B.C., Canada. E-mail: [cjlim@mail.ubc.ca](mailto:cjlim@mail.ubc.ca)

**Running Title:** *CD47-ligation induced cell death.*

**Key words:** CD47; cell death; apoptosis; Mcl-1; NOXA; honokiol

***Supplemental Figure Legends***

**Supp Fig 1. Flow cytometry assessment of cell death induced with CC2C6.** A sample scatter plot of PI vs Annexin V of WT and CD47^-/-^ Jurkat cells untreated or treated with 125ng/mL CC2C6 for 2hrs.

**Supp Fig 2. Generation of CD47^-/-^ cells and CD47-isoform specific re-expression. (A)** Jurkat cells were transfected to express a CRISPR-Cas9 construct targeting exon 1 of CD47. As shown is the sequencing of WT and CD47^-/-^ (JC47 clone 2-4), showing a single nucleotide insertion (highlighted in red) occurring near the PAM motif. **(B)** The indicated cells were untreated or treated with 125ng/mL CC2C6 for 2hrs and cell death assessed by flow cytometry for Annexin V binding. Error bars are the standard deviation for n=3 replicates for each experiment. **(C)** Same as (B). JinB8 is an independently isolated CD47^-/-^ strain.

**Supp Fig 3. (A) Effects of serum on CC2C6-induced cell death.** WT Jurkat cells were treated with 125ng/mL CC2C6 for 2hrs in serum-containing (10% FBS/RPMI) or in serum-free (1% BSA/RPMI) media and cell death assessed by flow cytometry for Annexin V binding. Error bars are the standard deviation for n=3 replicates for each experiment. **(B) Receptor internalization assay following ligation with antibodies.** WT cells were untreated or treated with primary antibodies CC2C6, B6H12 or ITGA4 and incubated for up to 3 days (see methods). Fluorescence conjugated secondary antibodies were added to treated samples to assess non-internalized receptors, while both primary and secondary antibodies were added to untreated samples to assess total surface receptor expression. % Cell surface receptor expression was calculated as follows: 100*[(sample GMFI –background GMFI)/(maximum GMFI – background GMFI)]; where D0 samples were set at 100%. Error bars are the standard deviation for n=3 independently conducted experiments. **(C) CC2C6 re-stimulation of cell death following initial treatment.** WT cells were treated with CC2C6 for 24hrs, then re-stimulated with or without a fresh bolus of CC2C6 for another 2hrs, and compared with cells untreated or treated with CC2C6 for only 2hrs. cell death assessed by flow cytometry for Annexin V binding. Error bars are the standard deviation for n=6 replicates from two independent experiments.

**Supp Fig 4. Comparison of chemotherapeutic susceptibility of WT and CD47^-/-^ cells.** The indicated cells were incubated with **(A)** Honokiol for 24hr, **(B)** doxorubicin for 48hr, or **(C)** vincristine for 48hr, at the indicated concentrations. Cell death was determined by flow cytometry using Annexin V binding.

**Supp Fig 5**. Comparison of CC2C6-induced cell death in various T-ALL cell lines. **(A)** The indicated cells were incubated with 125ng/mL CC2C6 for 2hr and cell death assessed by flow cytometry using the Annexin V binding assay. **(B)** The indicated cells were incubated with 125ng/mL CC2C6 alone or in combination with 30μM Honokiol for 24hr. Cell death assessed by flow cytometry using the Annexin V binding assay. Plotted is the averaged value of n=2 replicates of a representative experiment.

**Supp Fig 6**. Jurkat cells were incubated with 125ng/mL CC2C6 for the indicated times or with 40μM Honokiol for 4hr, prior to addition of the Caspase-Glo 3/7 reagent mix. The luminescence-based reporter for caspase activity was measured on a plate reader. Error bars represent the standard deviation for n=3 replicates.
